# Supplementary figures and images for: A comprehensive data-driven model of cat primary visual cortex
Source: PLoS Comput Biol. 2024 Aug 21;20(8):e1012342. doi: 10.1371/journal.pcbi.1012342 (PMC11371232; doi:10.1371/journal.pcbi.1012342)

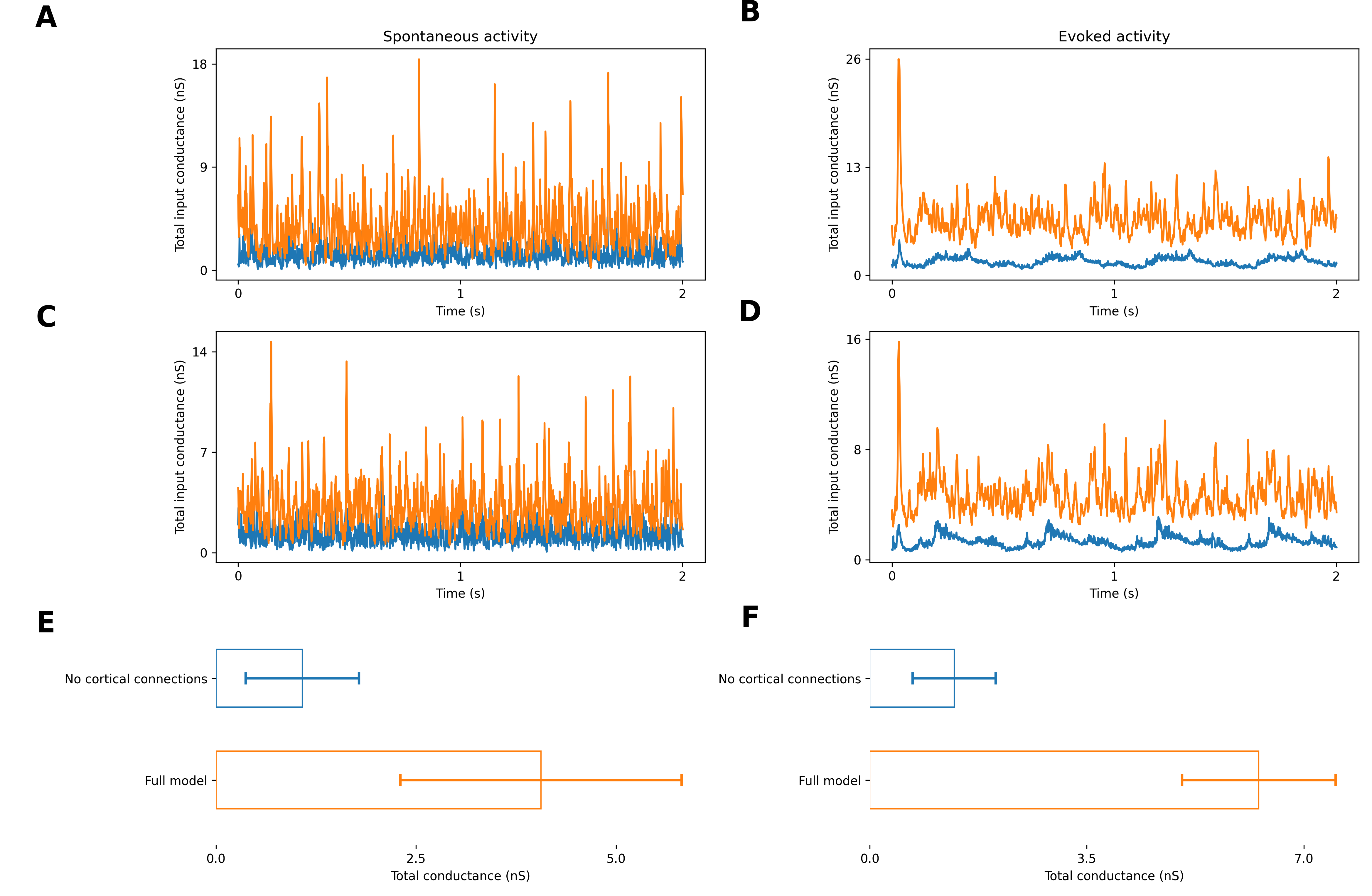

Supplement: S1 Fig — (A) Total synaptic conductance for an example Layer 4 excitatory cell during spontaneous activity in our V1 model (orange) and in a version containing only the thalamo-cortical connections to Layer 4 (blue). (B) Same, for evoked activity in response to a high contrast drifting grating with an orientation similar to the preferred orientation of the cell, averaged over 10 trials. (C) Same as (A), but for an example inhibitory neurons of the Layer 4 of the model. (D) Same as (B), but for an example inhibitory neurons of the Layer 4 of the model. (E) Layer 4 average total synaptic conductance for 40 seconds of spontaneous activity, for both our V1 model (orange) and the version with only cortico-thalamic afferents (blue). (F) Same as (E), for a subpopulation of cells that have corresponding preferred orientations, and for evoked activity in response to a high contrast drifting grating with an orientation similar to the preferred orientation of the cells, averaged over 10 trials. (PNG) [file pcbi.1012342.s001.png]

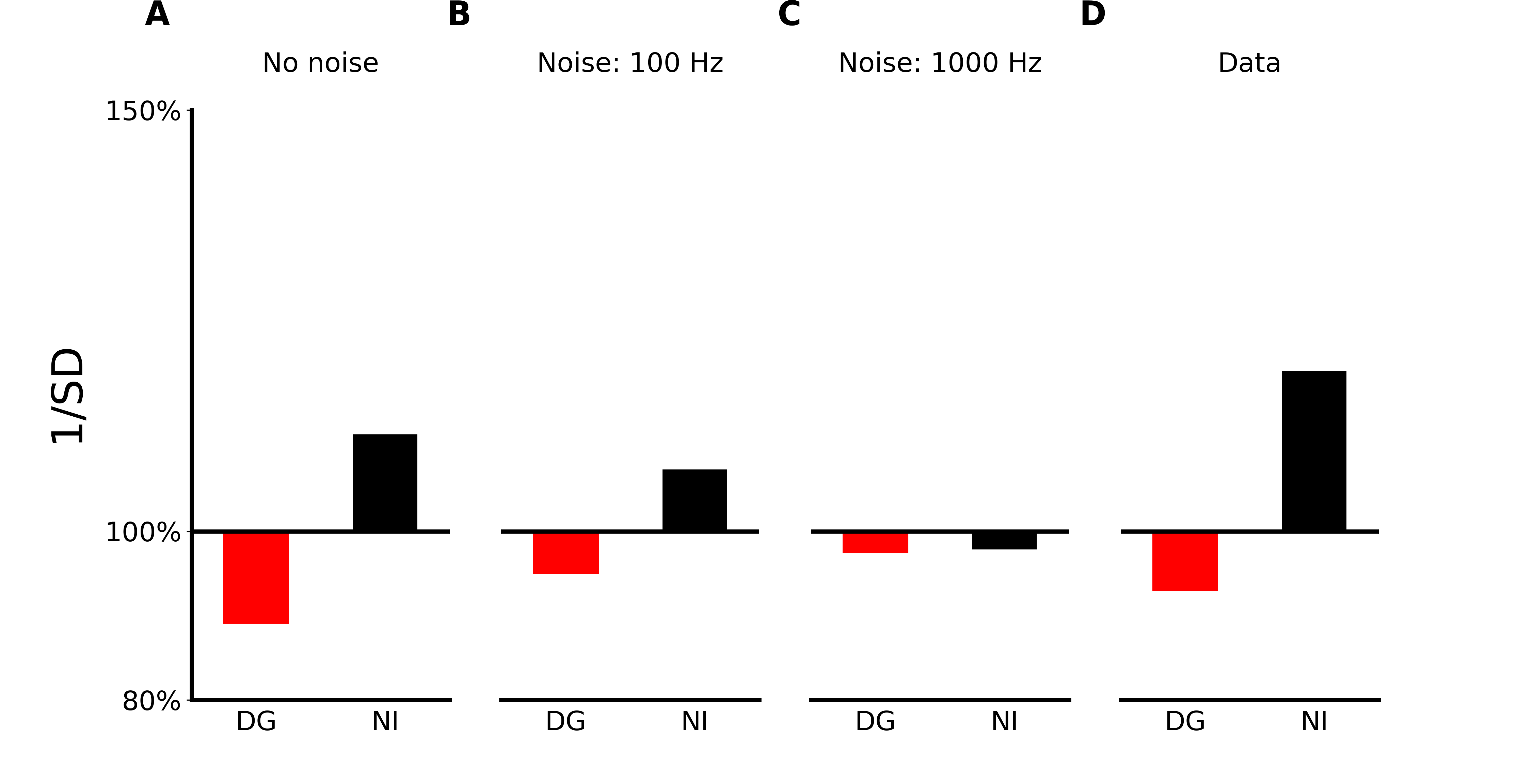

Supplement: S2 Fig — Results are averaged across all recorded excitatory neurons pooled across the two model layers. (A) For our V1 model with no external noise other brain areas. (B) For a modified version of our V1 model with 100 Hz of external noise input to every cortical neuron, with the weight of synapses formed by external connections to cortex set to 0.9 nS, and the weight of synapses formed by connections from Layer 4 to Layer 2/3 set to 0.9 nS. (C) Same as B with 1000 Hz of external noise, with the weight of synapses formed by external connections to cortex set to 0.65 nS, the weight of thalamo-cortical connections set to 0.675 nS and the weight of synapses formed by connections from Layer 4 to Layer 2/3 set to 0.65 nS. (D) Experimental results in V1 of the anesthetized cat [21]. (PNG) [file pcbi.1012342.s002.png]

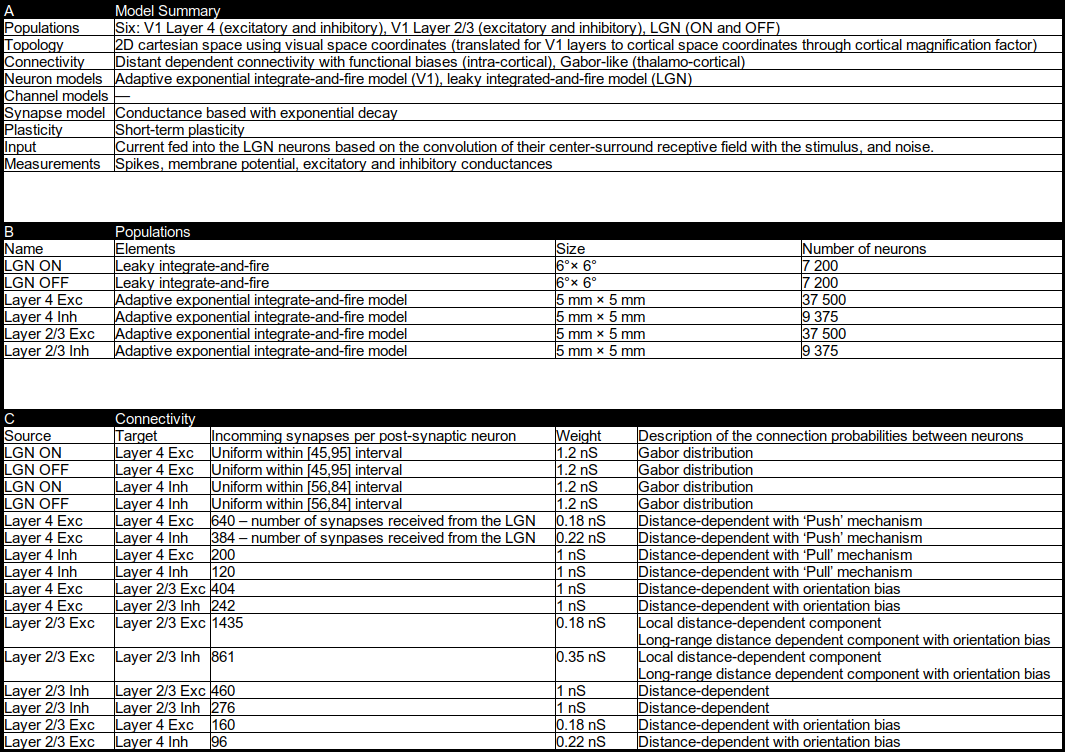

Supplement: S1 Table — The model is summarized in panel A and described in more details in panels B-G. See S2 Table for panel D, S3 Table for panel E, S4 Table for panel F, and S5 Table for panel G. (PNG) [file pcbi.1012342.s003.png]

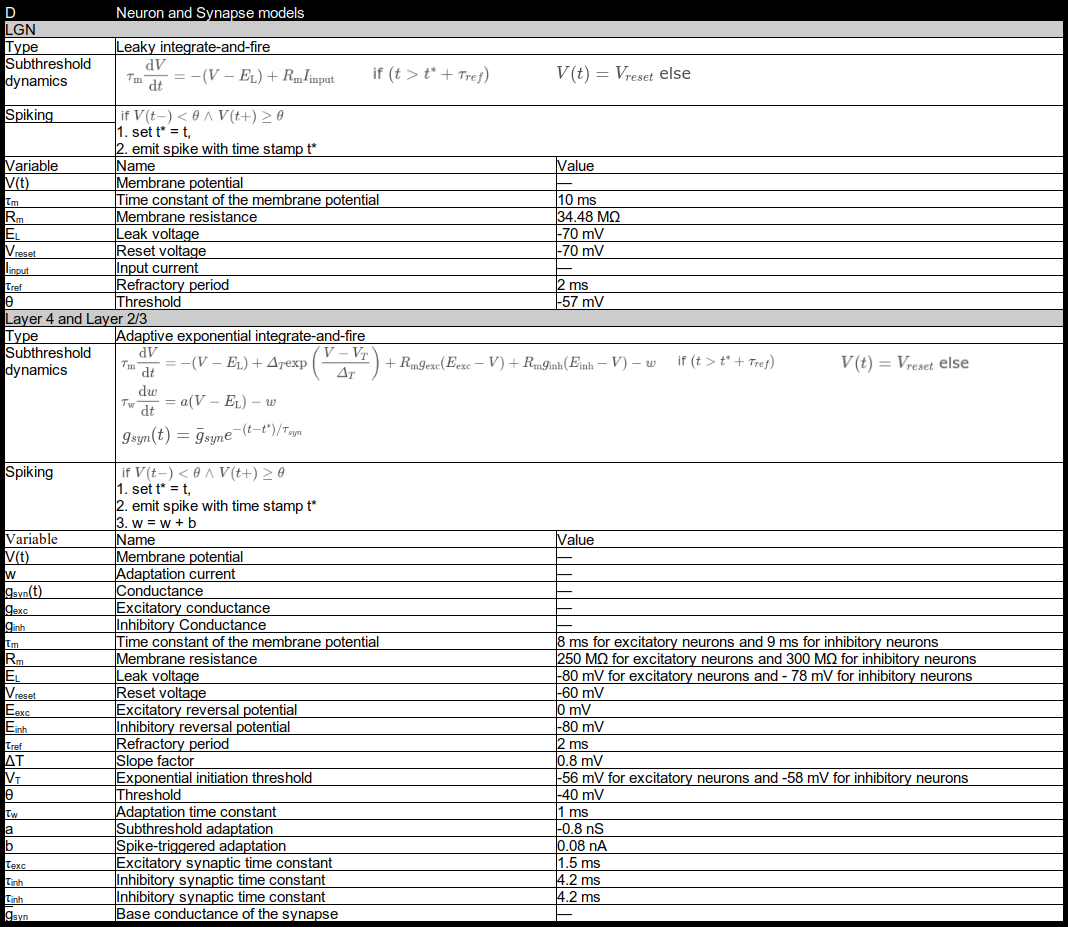

Supplement: S2 Table — See S1 Table for panels A-C, S3 Table for panel E, S4 Table for panel F, and S5 Table for panel G. (PNG) [file pcbi.1012342.s004.png]

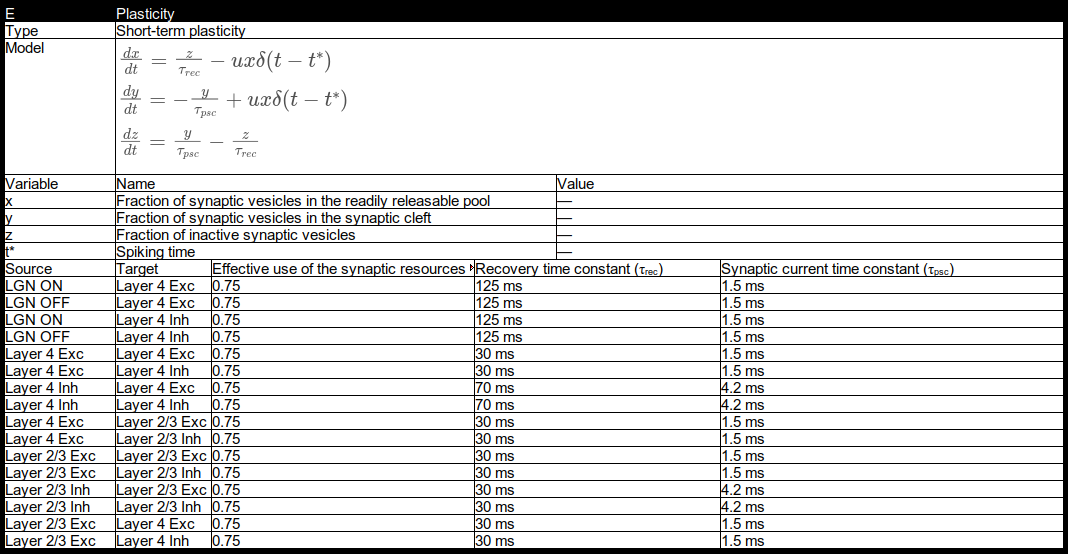

Supplement: S3 Table — See S1 Table for panels A-C, S2 Table for panel D, S4 Table for panel F, and S5 Table for panel G. (PNG) [file pcbi.1012342.s005.png]

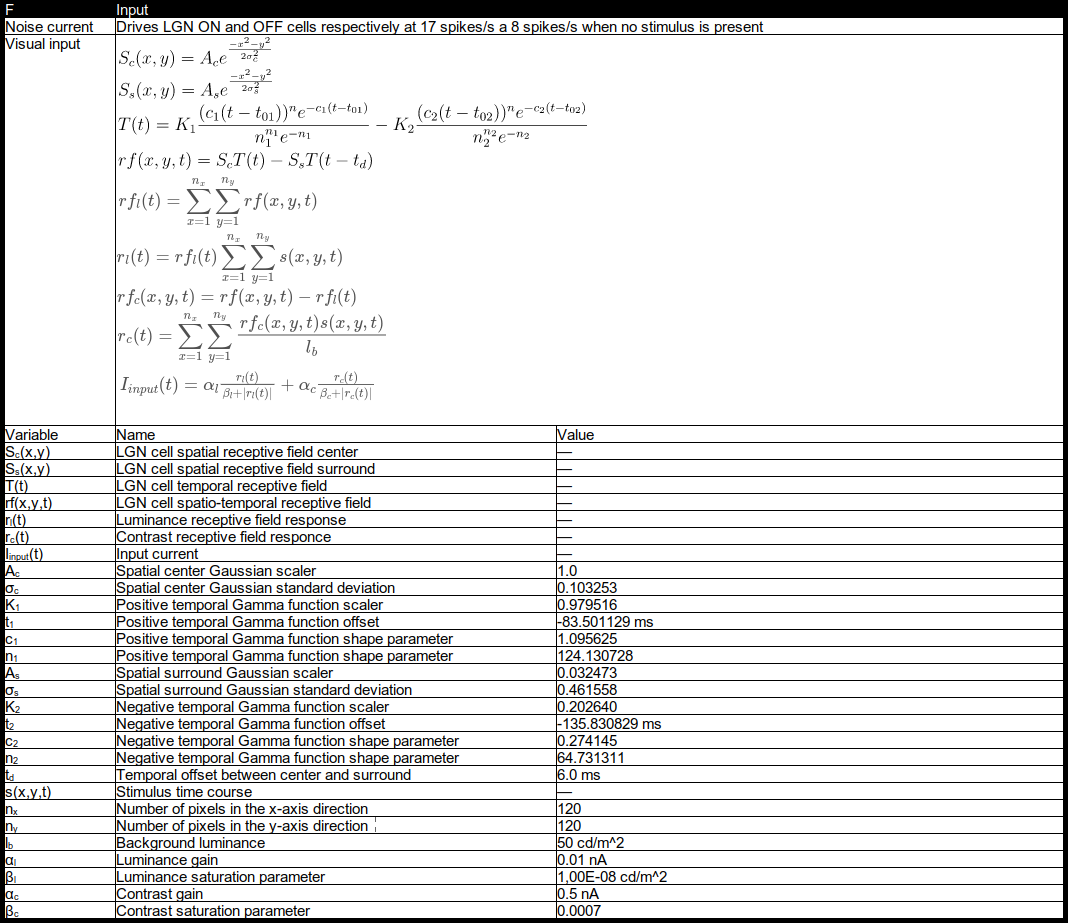

Supplement: S4 Table — See S1 Table for panels A-C, S2 Table for panel D, S3 Table for panel E and S5 Table for panel G. (PNG) [file pcbi.1012342.s006.png]

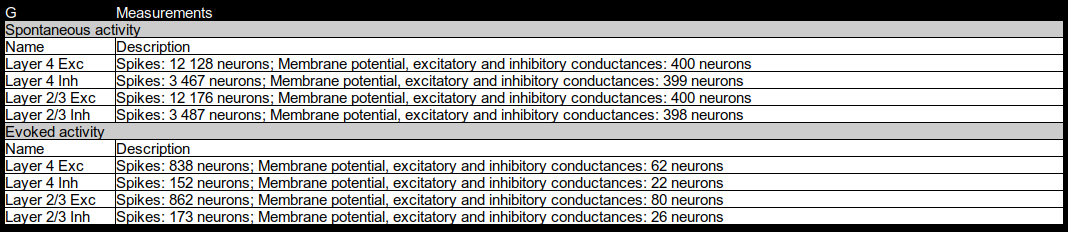

Supplement: S5 Table — See S1 Table for panels A-C, S2 Table for panel D, S3 Table for panel E and S4 Table for panel F. (PNG) [file pcbi.1012342.s007.png]
