# Supplementary material for: A comprehensive data-driven model of cat primary visual cortex
Source: PLoS Comput Biol. 2024 Aug 21;20(8):e1012342. doi: 10.1371/journal.pcbi.1012342 (PMC11371232; doi:10.1371/journal.pcbi.1012342)
Supplement: Attachment — Submitted filename: Response to reviewers.pdf [file pcbi.1012342.s008.pdf]

Our responses to reviewers' comments are in red. Changed text in the manuscript cited in our responses is in *italics*.

Reviewer #1:

We thank the reviewer for his very detailed and knowledgeable feedback and for the many suggestions that will certainly help us to clarify the model and better justify some parametric choices. We insert below our replies in the text body of his review.

General

* It would aid the understanding of the model specification if the authors could provide tables in the style of Nordlie et al. 2009, especially for connectivity and single-neuron parameters. It should also be specified if self-connections are allowed.

We have added these tables (Table S1-5) in the supplementary material. We have also added the following sentence at lines 787-790: *"In addition to the detailed description of the model provided in this section, we have also included an overview of the model's specification with tables following the template proposed by Nordlie et al. (Nordlie et al. 2009) (Table S1)."*

Our revised version now specifies that self-connections are allowed.

* In several places, the PSTH is used for ongoing activity, seemingly not aligned to a stimulus. Perhaps this would be better just called 'binned spike trains', 'spike histogram', or similar.

We have now replaced every occurrence of PSTH for ongoing activity with 'spike histogram'.

* The authors should discuss limitations due to the downscaling of the numbers of neurons and synapses with respect to real brains. With the assumptions of 50% local connectivity (which is rather low) and 50% failure rate, one still arrives at 1450 synapses per neuron, of which only 1000 are modeled.

We agree that it is possible that the true number of intra-areal synapses is larger than in the model, due to the underestimation of local connectivity or overestimation of synaptic transmission failures and we have hence added the following statements to the discussion to acknowledge this:

Lines 634-638

The model makes five main simplifications: (i) the omission of layers 5 and 6 (Thomson et al. 2010,Thomson et al. 2003), (ii) omission of the cortico-thalamic feedback pathway (Wang et al. 2006, Sillito et al. 1994), (iii) omission of the feedback from higher cortical areas (Angelucci et

al. 2003, Budd et al. 1998), (iv) reduction of neural dynamics to only two types (excitatory and inhibitory) (Xu et al. 2009, Nowak et al, 2003), and (v) ***the reduced cortical neuron and synaptic densities.***

Furthermore, we have discussed in detail the consequences of several interrelated modeling choices including the downsampling of modeled neuron count and connection numbers in a new discussion paragraph:

Lines 655-669
The need to model a large enough V1 surface to cover sufficiently long-range lateral interactions and contextual modulation, while keeping the computational complexity of the model manageable (to allow the simulation of experiments lasting thousands of seconds of biological time), lead to important compromises. We have decided to model only roughly one-tenth of neurons present in the cortical layers covered by our model, while the 1000 synapses modeled for layer 4 neurons and 2300 for layer 2/3 neurons potentially under-estimate the true number of local connections even if transmission failures are factored in (see Materials and Methods). In addition, connections coming from outside of V1 were not modeled at all. While the results presented in this study show that the lower neural density is not a limiting factor for the emergence of the range of phenomena addressed here, previous computational studies showed that the neural density can have an important impact on the correlation structure of the network activity (van Albada et al. 2015), or impact the statistics of spontaneous cortical waves (Davis et al. 2021). It is thus possible that, as we seek to explain an ever greater range of phenomena in the future, we will have to re-evaluate the neural density down-sampling.

Regarding the exact synaptic count: in the model layer 2/3, as stated in the manuscript in lines 856-859, we simulated 2300 synapses per L2/3 neurons while only 1000 in L4 neurons. This was motivated by the observations that layer 4 has a less dense recurrent connectivity than layer 2/3 (Binzegger et al. 2004, Izhikevich et al. 2008). The estimate of the number of synapses per V1 neuron (5800) is the average across V1 neurons (as far as we are aware no layer resolved synaptic counts are available). If we average the 1000 and 2300 we get 1650 synapses, which is in fact greater than the estimate of 1450 the reviewer is pointing out (which excludes inter-areal connections and compensates for synaptic transmission failures). Nevertheless, these numbers remain rough estimates, hence our concern was to only be in a qualitative agreement with these estimates.

* The classification into simple and complex cells does not consider the main properties that distinguish these cells types: larger receptive fields of complex cells and a lack of clear on and off regions for complex cells.

As described in our manuscript, the classification into simple and complex cells is based on the ratio between the fundamental component F1 and the DC component F0 of the Fourier transform of the spike histogram of the cells in response to an optimally oriented drifting grating.

The fact that complex cells have a strong F0 component and a weak F1 component is a direct consequence that their receptive fields lack clear segregation between on and off subfields, as their response to an optimal drifting grating would lack a phasic component. To put it another way, cells that we classify as complex cells, have to have overlapping ON-OFF subregions, otherwise they would be classified as simple by the Fourier based method.
This is a standard classification method for the simple/complex subtypes that has been widely adopted in a range of previous studies (Skottun et al. 1991, Priebe et al. 2004, Scholl et al. 2013), and that has been shown to correlate with the overlap of the subregions (Mata et al. 2005). We consider this a more robust classification method than one relying on identification of on-off subregions, (i) often done manually with flashed light and dark stimuli, or (ii) using reverse correlation during sparse or dense noise mapping. The latter depends on an arbitrary threshold for determining the edge of the individual on-off subregions, which in turn depends on the amount of data collected for the reverse-correlation. They also depend on the noise statistics (sparse or dense) used to map the receptive field (Fournier et al., 2011). Perhaps most importantly, the reference data on simple/complex classification that we compare our data to have used the fourier based classification method. For all these reasons we advance that the simple/complex classification procedure in our study is adequate.

Regarding the size of the receptive fields of simple vs. complex cells, we haven't found any clear data in cat showing that complex cells have larger receptive fields than simple cells, that control for biases due to laminar differences in receptive field sizes. We therefore prefer to focus on the laminar differences in receptive field sizes, where it has been shown that Layer 2/3 neurons having smaller receptive fields than Layer 4 neurons (Jones et al., 2000), which our model replicates and that we have reported on in subsection 4.3.

* The README in the repository https://github.com/CSNG-MFF/mozaik-models/tree/main/LSV1M is rather minimal and should contain an overview of the files included in the repository and what each file is for. I was for instance not able to identify the precomputed orientation map that was used. There are two candidates with apparently different resolutions (or_map_new_16x16 and or_map_new_6x6) but the documentation is missing. A file whose name suggests it is a main file, run.py, appears to be about a different model, that of Vogels and Abbott. Furthermore, the required software/package versions should be mentioned.

Thank you for pointing out the lack of instructions for running the model, principally because the Mozaik-models repository didn't refer at all to the installation of our Mozaik simulation workflow. We have now added a sentence in the README header, which specifies that, in order to run the model, one should first install Mozaik and its dependencies. It also includes an explicit link to the Mozaik repository (https://github.com/CSNG-MFF/mozaik) for more detailed installation instructions.

We have also added the following sentence in the revised version of the manuscript (lines 761-765):

“The model can be run by first installing Mozaik and its dependencies as indicated in the Mozaik Github repository, and then by downloading the implementation of model at https://github.com/CSNG-MFF/mozaik-models/ [version 1.0] and following the instructions to run each experiment.

In addition to instructions on how to run the model, the README file of the mozaik-models repository now also contains an overview of the files it contains as rightfully suggested.

We thank you for pointing out that the run.py file refers wrongly to the implementation of the Vogels and Abbott model, that is an error on our side. We have now corrected it so that it clearly refers to the preprint of the manuscript.

* The figures have very low resolution; presumably this would be fixed if the paper is accepted.
According to our communication with PLOS support, the figures in the system-built PDF are intended as previews of the figures and appear at a lower resolution so that it is easier for editors and reviewers to download the manuscript file.

So the figures will indeed have a higher resolution in the final version of the article if it is accepted.

Introduction

l. 43: "Thalamocortical connectivity in Layer 4 follows a push-pull organization [...] .": While the push-pull connectivity is mediated by thalamocortical projections, it is itself a characterization of the intra-cortical connectivity within L4 of V1, and not of the thalamocortical connectivity targeting Layer 4. The above sentences makes this sound otherwise.

Thank you for noticing this imprecision. We replaced 'thalamocortical' by 'intra-laminar', and made further changes in the sentence to specify that the second part refers to the distance-dependent connectivity:
"*Intra-laminar connectivity in Layer 4 follows a push-pull organization (Troyer et al. 1998) (Fig 1E), and the distance-dependent intra-cortical connectivity follows parameters derived from experimental data (Binzegger et al. 2004, Stepanyants et al. 2008, Buzás et al. 2006)(Fig 1AB).*"

Results

l. 75: "The only external source of variability in the model is a white noise current injection into lateral geniculate nuclesu (LGN) cells which induces their spontaneous firing rate at about 17 Hz for ON cells and 8 Hz for off cells [...]".
As a part of the cortex, the modeled patch would not only receive input from parts of V1 not modeled, but also from other cortical areas. Especially the foveal region in the area centralis is known to be strongly targeted by feedback projections, at least in macaque V1 (Wang et al. 2022, Retinotopic organization of feedback projections in primate early visual cortex). While

modeling other parts of the primary visual cortex not represented in the given model (other layers for example, or areas establishing feedback projections to V1) is clearly beyond the scope of the presented work, they still could be provided as stochastic 'background input'. Why is this not done?

We have added the following two paragraphs into the discussion that address the reviewers question:

“The absence of a significant portion of the incoming connections in our model (primarily due to omission of extra-V1 innervation) raises several issues. To properly model all the connections coming from extra-V1 areas would require us to model all those source areas, which is outside of the scope of this study. This however raises the question of how to compensate for the absence of the drive from the missing connections. A common solution is to model an extra independent Poisson spiking input into each cortical neuron (Schmidt et al. 2018), that compensates for the missing drive. This is quite reasonable when the rate of such compensatory input can be estimated as approximately Poissonian (e.g. spontaneous condition). However, for visually evoked conditions, where the magnitude of the external drive might depend on the origin of its source, the ongoing visual stimulus and likely even the specific function of the receiving V1 neuron, constructing a reasonable approximation of an extra-areal drive is extremely difficult. Furthermore, it is reasonable to expect that the visual stimulus heavily modulates the statistics of the stochastic extra-areal input, making an approximation with a stationary Poissonian input inappropriate. This latter issue is particularly problematic given that we wished to study the modulation of fine temporal statistics in the spontaneous vs. visual driven conditions.

To explore quantitatively the latter issue, we have repeated the experiment performed in section 4.4 that compares the trial-to-trial variability of the membrane potential in response to drifting sinusoidal grating vs. naturalistic stimulus (Fig 10), but this time in a model enriched with an external Poissonian input impinging on all cortical neurons at a rate of 1000 Hz (Fig S2C), which corresponds to a roughly 1 Hz spontaneous rate arriving via 1000 extra-areal connections. As can be seen, unlike in the original model (Fig S2A), the model with external input shows virtually no change of trial-to-trial variability for both the grating and naturalistic conditions in contradiction to the data (Fig S2D). This is because the constant, stimulus independent, external Poissonian noise drowns the stimulus dependent modulation of variability within the cortico-thalamic network.

For all these reasons our decision was to not include such compensatory external inputs in our model. Yet, as we show in section 4.1 and 4.2, our model reaches realistic levels of spontaneous and evoked activity at both the spiking, membrane potential and conductance levels. How is this possible? As we explain in section 6.1.6, the existing evidence on the synaptic strength of the various projections included in our model is very limited, leaving a lot of leeway for setting the exact synaptic weights. To deal with this unknown we set the synaptic weights to achieve an overall balance between excitation and inhibition that supports

reasonable levels of both spontaneous and evoked activity, while being compatible with the limited physiological findings. It is thus possible that these broad constraints on synaptic weights allowed us to compensate for some of the external drive into stronger-than-biological synaptic weights. This points to a general limitation of reductionist modeling where missing aspects of the model are often (at least partially) absorbed into unrelated free parameters of the model.

l. 80: "Due to the low proportion of synapses of thalamic origin in the model cortex, the variability of spontaneous and visually evoked cortical activity is largely driven by internal cortical dynamics."
This is asserted without evidence. Surely, since neurons in L2/3 do not receive external input from outside the model this is true trivially. But for neurons in L4 one can do the following back-of-the-envelope calculation:
In the model, L4 excitatory cells have 1000 recurrent and around 140 TC synapses. Let's assume that 50% of synapses are excitatory, 50% inhibitory (probably there are more excitatory).
Spontaneous firing rate for excitatory neurons in L4 is 1.4 Hz, in L2/3 2 Hz. The weight for L4E->L4E connections is 0.18 nS, for L2/3E->L4E 1 nS.
The latter connections are 20% of the synapses formed there, so 200 per cell.
This implies that the incoming recurrent input per excitatory neuron from all excitatory neurons is about (omitting units) 500 * 0.8 * 1.4 * 0.18 + 500 * 0.2 * 2 * 1 = 100.8 + 200 = 300.
The inhibitory firing rate is 7.6 spikes/s in L4, 4.7 spikes/s in L2/3. The inhibitory weights are set to 1 nS.
This results in incoming recurrent input per excitatory neuron from inhibitory neurons (again omitting units, ignoring different signs from excitatory and inhibitory neurons) 500 * 0.8 * 7.6 * 1 + 500 * 0.2 * 4.7 * 1 = 3040 + 470 = 3500.
There are 140 TC synapses per neuron firing at a rate of about 12 spikes/s with a weight of 1.2 nS leading to an input of about 140 * 12 * 1.2 = 2016.
The fraction of TC input to excitatory cells now is 2016 / (3500 + 300 + 2016) = 0.35.
This fraction gets higher if there are relatively more excitatory recurrent connections, so that it may actually exceed 0.5. Furthermore, synchronization plays a role in determining which connections are more influential. It is thus unclear if indeed "variability of spontaneous and visually evoked activity is largely driven by internal cortical dynamics."

It is indeed correct that the thalamo-cortical drive plays a non-negligible role in the dynamics of Layer 4 despite the low number of thalamo-cortical connections, but what we were emphasizing here is that the internal cortical connectivity has a higher impact. We are thankful and impressed that you took time to make these calculations, but your argumentation, as detailed as it is, doesn’t take into account several other factors such as inhibitory synapses having a longer time constant than excitatory synapses or that the thalamo-cortical synapses display more short-term depression than the other synapses of the model due to their larger recovery time constant.

We believe that looking at the average synaptic conductances of the neurons in a version of the model without any intra-cortical connections and comparing it to the average synaptic

conductances in the Layer 4 neurons of the full model would be a better indicator of the impact of the thalamo-cortical connections in the model's dynamics. This measure doesn't take into account the synchronization of the inputs, but this factor is minimised for spontaneous activity as the input to every LGN cell is independent noise, and cortical neurons have a low synchrony in this condition. In response to drifting gratings, the average synaptic conductances of the neurons selective to the orientation of the grating fall down to 20% of their original value when we disable every cortico-cortical connection. For spontaneous activity, this value reaches 30%. This supports us in stating that the activity of the model is largely driven by internal cortical dynamics.

We have now added this figure as supplementary figure to the paper, and refer to it in the statement claiming cortical dominance:
"Due to the low proportion of synapses of thalamic origin in the model cortex (Da Costa et al. 2011) the variability of spontaneous and visually evoked cortical activity is largely driven by internal cortical dynamics (Figure S1)."

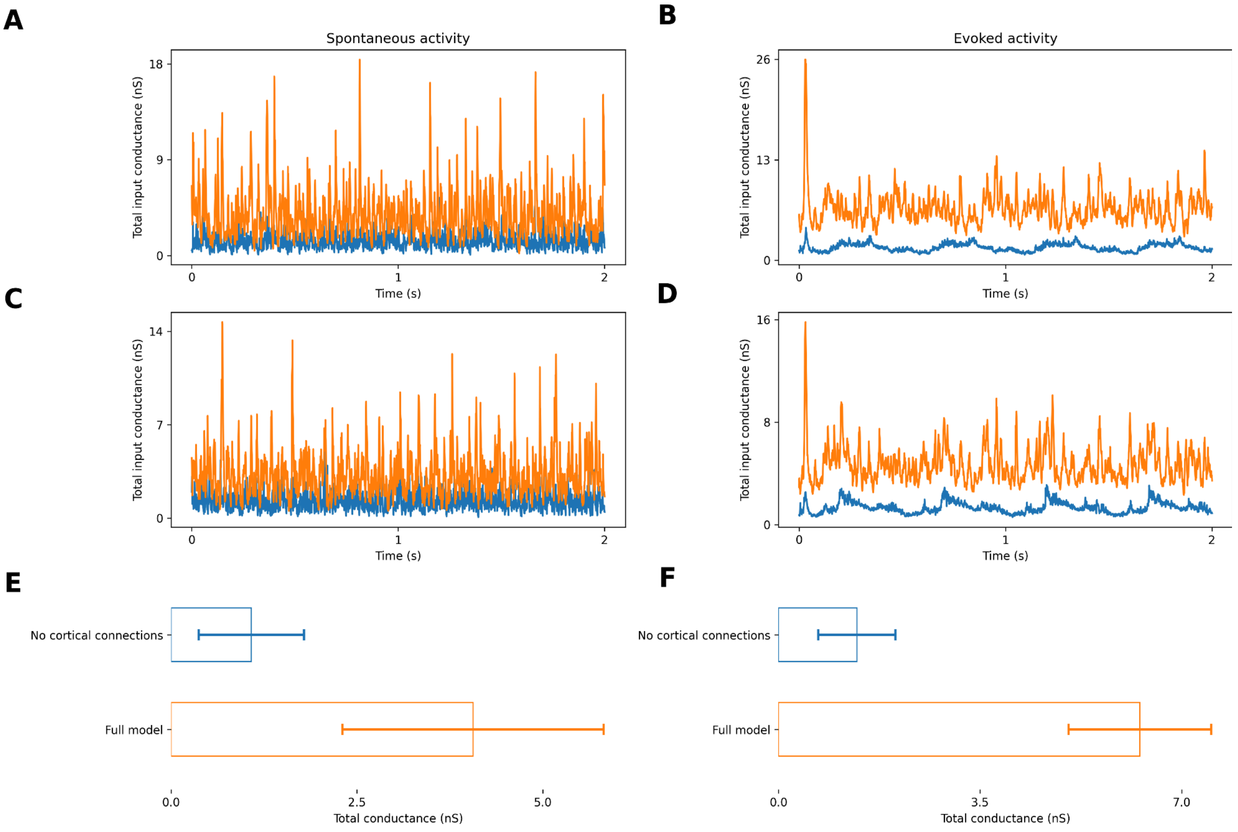

Fig S1. Decrease of synaptic input in Layer 4 in the absence of cortico-cortical connections . (A) Total synaptic conductance for an example Layer 4 excitatory cell during spontaneous activity in our V1 model (orange) and in a version containing only the thalamo-cortical connections to Layer 4 (blue). (B) Same, for evoked activity in response to a high contrast drifting grating with an orientation similar to the preferred orientation of the cell, averaged over 10 trials. (C) Same

as (A), but for an example inhibitory neurons of the Layer 4 of the model. (D) Same as (B), but for an example inhibitory neurons of the Layer 4 of the model. (E) Layer 4 average total synaptic conductance for 40 seconds of spontaneous activity, for both our V1 model (orange) and the version with only cortico-thalamic afferents (blue). (F) Same as (E), for a subpopulation of cells that have corresponding preferred orientations, and for evoked activity in response to a high contrast drifting grating with an orientation similar to the preferred orientation of the cells, averaged over 10 trials.

Fig. 3B-E: The red traces are not sufficiently clearly visible. The same holds for some of the red traces in Fig. 5.

As mentioned before, the resolution of these figures will be higher in the final version of this paper if it is accepted. Nevertheless, we modified the Figure 3 such that the excitatory and inhibitory conductances traces are now shown separately in different panels. We didn't do the same for Figure 5, as we consider that the excitatory conductances are sufficiently visible when the resolution of the figure is high enough, and find it important to plot them stacked together to show the inverse relationship between excitatory and inhibitory conductances in Layer 4 of the model for optimal gratings.

l. 85: In panels A-F of Figure 4, the authors always plot quantities for L2/3 neurons above the same data for L4 neurons. In panel G, however, neurons in L4 are above L2/3. This then is consistent with Figures 5, 6, 7, 8, but not with the raster plot. Please make this consistent to increase understandability.
This lack of consistency is indeed unhelpful. We have modified Figure 3 and 4 so that Layer 4 is shown on top of Layer 2/3 on every panel. Thank you for spotting this.

l. 90-91: It would be better to compare with awake recordings.

Although we specified in the discussion that this model is a model of anesthetized cat V1, we acknowledge it could have been more clear and therefore added this information in the introduction (lines 32-34) as well:
“here we offer a basis for such an integrationist effort: a detailed, biologically realistic model of anesthetized cat V1”.

l. 97: It is very selective to compare with a study that reports higher spontaneous firing rates in L2/3 compared to L4. Many studies show the opposite (e.g., Gur et al., Cerebral Cortex 2005 for macaque V1; de Kock and Sakmann PNAS 2009 for rat S1; see also Potjans & Diesmann 2014).

We apologize for the following mistake in our manuscript: We wrote that Kim et al.,2016 reported higher spontaneous rate in L2/3 compared to L4 in macaques while the study was made on anesthetized cats.

Moreover, Silitto et al.,1977 report higher firing rates in complex cells than in simple cells in anesthetized cat V1. As simple cells dominate L4 and complex cells are more numerous in L2/3, we believe this study also strengthens our claim.

We thank you for providing other articles reporting laminar differences in spontaneous activity, but as the first one studies awake macaque and that the second one studies awake rat S1, we don't consider they contradict our claims about anesthetized cats V1 as this might reflect differences across species (see Frégnac and Bathellier, 2015), cortical areas, and anesthetized state VS awake state.

l. 142: Please add references on experimental studies showing steady depolarization in L2/3 neurons in response to optimally oriented gratings.

We added two references showing that complex cells membrane potential depolarizes steadily in response to optimally oriented gratings: Carandini & Ferster, 2000 and Contreras & Palmer, 2003.

l. 150: It seems that this is the first mention of Figure 6. It starts with panel C, not panel A. This is a bit confusing. It would be better to refer to panel A first or change the order of panels within the figure.

Thank you for pointing that out, we now refer to panels A and B in the first sentence of the paragraph to fix this.

Figure 6 (correctly) uses sp/s as the unit for the firing rate, whereas in other places, 'Hz' is used, which is actually reserved for processes with a fixed frequency.

We modified the text as well as Figure 4 so that only ‘sp/s’ appears.

Fig. 6: It is strange that the integrals of the histograms in corresponding lines of panels C and D of Fig. 6 do not amount to the same numbers of neurons. It seems these panels do not actually share the same y-axis.

You are indeed correct, we have now added a separate y-axis for the plots in panel D. We have also slightly moved down the labels of the x-axis of these plots so that they align better with the adjacent plots.

Figures 6 and 7: It is highly unrealistic to have lower contrast leading to greater responses (see for instance Albrecht and Hamilton J Neurophysiol 1982; Reich et al. J Physiol 2000). I suspect the legends are reversed (as also suggested by the Fig. 7 caption, which says the blue lines are for high contrast and the black lines for low contrast). Also please clarify if the low contrast curves in these figures are for 10% or for 15% contrast, as conflicting information is given (also

in Fig. 9). Finally, please mention in the Fig. 7 caption what is the meaning of the shaded areas, and make sure that the shading is consistently applied across all panels.

We apologize for the confusion with the contrast labeling in the figures. The contrasts in the legends were indeed reversed, and the labels should have always indicated 10% contrast. We have now corrected that. The shaded areas correspond to the standard error of the mean. We added this information in the captions of both Fig. 6 and Fig. 7.

l. 190: "To assess to what extent this idealized scheme is true in this model, we have plotted the orientation tuning curves of the mean (DC) and first harmonic (F1) components of the sub-threshold signals across the different neural types and layers in Fig 7." Here "DC" is used as abbreviation, whereas the figure and caption use "F0". Please harmonize the notation here. Also, please clarify how F1 is computed.

We replaced DC with F0 every time DC was used in this context. To follow the same logic of harmonization, we also replace 'V1/V0' in line 1140 by 'F1/F0'.
We have also now added the following sentence in the methods in lines 1135-1136 to clarify how the F1 is computed: *"The F1 component was defined as the Fourier component corresponding to the temporal frequency of the DG stimulus".*

l. 245-246: Please give units for the F1 components.

It was fixed.

Figure 9: Please add units to horizontal axes at lowest plot in panel A, B, C.
Panel G: What is CRF? Classical receptive field? I think the abbreviation is not introduced.

We added the label "Diameter (°)" on the x-axis of these plots. We replaced "CRF" by "MFD" as we use this abbreviation (standing for Maximum Facilitatory Diameter) in the manuscript.

l. 256: "While most excitatory neurons in both cortical layers show surround suppression [...]." This seems not to be quantified. In the caption of Figure 9 it says that traces of typical cells in L4 and L2/3 are shown. How many cells are "typical"?

We believe that this is quantified in the panel D of Figure 9, which shows the suppression index of cells from both cortical layers. This panel shows that in majority excitatory cells in the model have a non-zero suppression index which indicates that they display surround-suppression. It also shows that Layer 4 cells can have different size-tuning profiles ranging from no suppression to moderate suppression, as illustrated by the two Layer 4 example cells in panel A. Finally, this panel highlights that the vast majority of Layer 2/3 excitatory cells display moderate surround-suppression as illustrated by the Layer 2/3 example cell in panel A.

l. 284: Instead of the central eccentricity, it makes more sense to compare with the average eccentricity of the model here (which will also be smaller than the ones in the experiments).

For the size tuning protocol we only analyzed the responses of neurons located within a 0.4° radius from the center of the model, as written in the methods in subsection 6.3.2. The average eccentricity of these neurons is therefore similar to the eccentricity at which the model is centered.

Figure 10: Figure 10B is referenced before 10A. It would be better to swap them. It would also be interesting to see the same plot for L2/3.

Thank you for noticing that. We modified the orders of the A and B panels to respect the order of reference in the text. We have also enlarged the figure to include an example cell from Layer 2/3, and have modified the panels numbering and the caption accordingly.

l. 327: "slightly higher": Here, 0.63 is called "slightly higher" than 0.441, whereas the previous sentence called 0.221 simply "higher" than 0.187. The former difference is greater in both absolute and relative terms; the wording should not try to mask this.

Indeed "slightly higher" was misleading in that sentence and therefore we have replaced it by “higher”.

l. 344: "The lack of direct external noise injected into cortical model neurons was crucial for achieving the results presented in this section." The authors state that another model of V1 could not reproduce the stimulus-dependent variability effect at the level of the membrane potential, albeit being able to do so on the level of the spiking activity. It is argued that this is due to the lack of external input noise injected into the model neurons. It seems plausible, however, that neurons in V1 receive input from the surrounding cortex as well as other cortical areas that might - at least to some degree - 'look' like noise. What is the justification for not adding this, apart from thereby obtaining the seemingly correct result? If this only works in the absence of noise, something is probably wrong.

In the simulations presented in this manuscript we construct an ‘isolated’ V1 cortical network that only receives stochastic response from the thalamus and expresses stochastic asynchronous irregular population responses during spontaneous activity. We then demonstrate that the trial-to-trial variability of such ‘isolated’ V1 network is strongly modulated in a stimulus dependent manner (Figure 10).

On the basis of these findings, a reasonable hypothesis is to assume that the trial-to-trial variability of other higher-level visual areas will be also strongly modulated by stimulus through the same mechanism, and hence cannot be reasonably approximated as a stationary Poissonian process. Surely, there is potentially also the input from non-visual areas , but we expect it to be a relatively small fraction of the total local and extra-areal input to V1. In summary, given our findings in an ‘isolated’ network, we do not consider a stationary Poissonian input as a self-consistent replacement of external input for visual evoked conditions in V1.

We have discussed this issue, in slightly less detail, in the discussion of the manuscript, also adding (and referring to) new supplementary figure (Figure S2) showing that adding a moderate (100 Hz) amount of Poissonian external input to our simulations does not qualitatively change the results regarding trial-to-trial variability of visually evoked responses. But addition of substantial (1000Hz) amounts of external Poissonian input (that would correspond to modeling of substantial proportion of extra-areal inputs as Poisonian inputs) virtually eliminates the stimulus dependent modulation of trial-to-trial variability as can be seen in figure S2 below. Note however that these observations do not invalidate the model, as our hypothesis, we justify earlier, postulates that external input from extra-V1 areas is itself modulated by the visual stimulus.

This is the added paragraph to discussion, and below is the new supplementary figure 2:

"The absence of a significant portion of the incoming connections in our model (primarily due to omission of extra-V1 innervation), raises several issues. To properly model all the connections coming from extra-V1 areas would require us to model all those source areas, which is outside of the scope of this study. This however raises the question of how to compensate for the absence of the drive from the missing connections. A common solution is to model an extra independent Poisson spiking input into each cortical neuron (Schmidt et al. 2018), that compensates for the missing drive. This is quite reasonable when the rate of such compensatory input can be estimated as approximately Poissonian (e.g. spontaneous condition). However, for visually evoked conditions, where the magnitude of the external drive might depend on the origin of its source, the ongoing visual stimulus and likely even the specific function of the receiving V1 neuron, constructing a reasonable approximation of an extra-areal drive is extremely difficult. Furthermore, it is reasonable to expect that the visual stimulus heavily modulates the statistics of the stochastic extra-areal input, making an approximation with a stationary Poissonian input inappropriate. This latter issue is particularly problematic given that we wished to study the modulation of fine temporal statistics in the spontaneous vs. visual driven conditions.

To explore quantitatively this issue, we have repeated the experiment performed in section 4.4 that compares the trial-to-trial variability of the membrane potential in response to drifting sinusoidal grating vs. naturalistic stimulus (Fig 10), but this time in a model enriched with an external Poissonian input impinging on all cortical neurons at a rate of 1000 Hz (Fig S2C), which corresponds to a roughly 1 Hz spontaneous rate arriving via 1000 extra-areal connections. As can be seen, unlike in the original model (Fig S2A), the model with external input shows virtually no change of trial-to-trial variability for both the grating and naturalistic conditions in contradiction to the data (Fig S2D). This is because the constant, stimulus independent, external Poissonian noise drowns the stimulus dependent modulation of variability within the cortico-thalamic network.

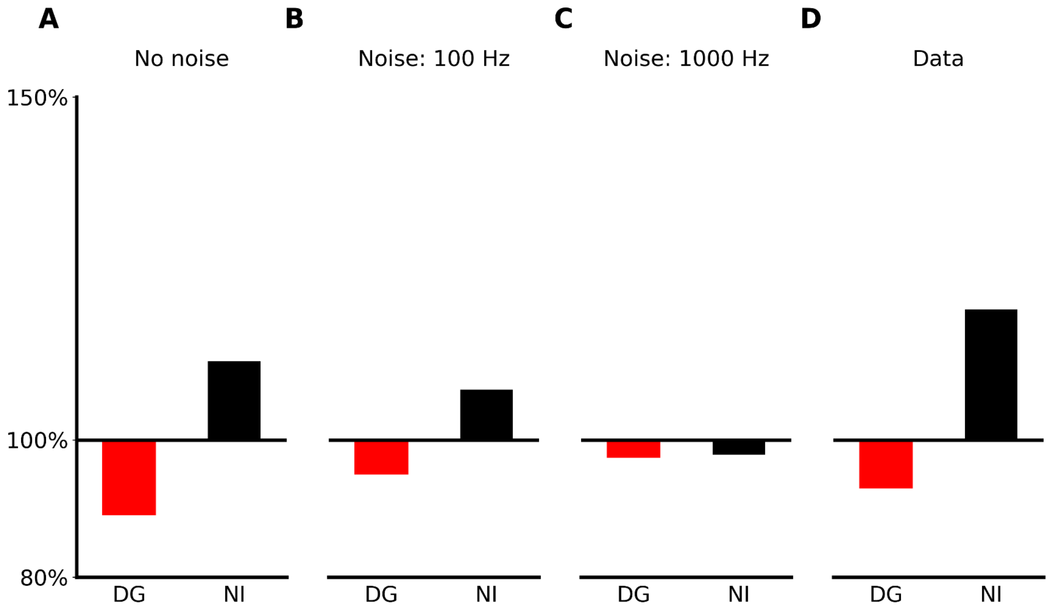

Fig S2. The inverse of the stimulus-locked time-averaged standard deviation of membrane potential (1/SD) across trials, averaged across cells, relative to the value for ongoing activity. Results are averaged across all recorded excitatory neurons pooled across the two model layers. (A) For our V1 model with no external noise other brain areas. (B) For a modified version of our V1 model with 100 Hz of external noise input to every cortical neuron, with the weight of synapses formed by external connections to cortex set to 0.9 nS, and the weight of synapses formed by connections from Layer 4 to Layer 2/3 set to 0.9 nS. (C) Same as B with 1000 Hz of external noise, with the weight of synapses formed by external connections to cortex set to 0.65 nS, the weight of thalamo-cortical connections set to 0.675 nS and the weight of synapses formed by connections from Layer 4 to Layer 2/3 set to 0.65 nS. (D) Experimental results in V1 of the anesthetized cat (Baudot et al. 2013).

l. 336: The formulation calling the inverse of a standard deviation a "variance" is not ideal.

We agree that calling it variance is misleading and therefore chose to call it 'stimulus-locked variability' instead. We have also reformulated the paragraph in lines 336-349 to better explain the measured quantities:

“Next, Baudot et al. (Baudot et al. 2013) found that relative to the trial-to-trial variability of membrane potential during spontaneous activity, the variability of membrane potential during DG stimulation increases, while it decreases during NI stimulation (Fig 10J). Baudot et al. quantified the variability modulation as the inverse of the stimulus-locked time-averaged standard deviation across trials (1/SD), and expressed as a percentage relative to the value for ongoing activity, meaning that values below 100% reflects an increase of trial-to-trial variability. The results of the same analysis in the model are shown in Fig 10(GH). In both model layers we observe an increase of stimulus-locked variability for DG (relative 1/SD equal to 91 ± 1.05% in Layer 4 and 88 ± 0.2% in Layer 2/3). For the NI condition, we observe a decrease of

stimulus-locked variability in Layer 4, as well as a small increase in Layer 2/3 (relative 1/SD equal to 130 ± 0.53% in Layer 4 and 97 ± 0.22% in Layer 2/3). However, note that in Baudot et al. (Baudot et al. 2013) the results are pooled across neurons of all layers, and the same treatment of our simulation data yields a good quantitative match to the experimental data (Fig 10I).”

Discussion

l. 382: It is not at all a given that neuroscience should converge upon a single model. Even if one considers only a single individual of a single species, models at different levels of resolution and including different biological aspects will continue to be useful for different purposes.

This text was unnecessarily philosophical. To remain ‘on topic’, we have removed the sentence (and ‘therefore’ from the following one).

l. 385: Similarly, it is not a given that all models should pass the same validation tests. First of all, experiments are not perfect and different experiments may even give contradictory results. Second, which validation tests a model should pass may depend on the purpose of the model.

As mentioned in the previous response this text was removed.

Table 1: I am not sure if this can easily fit, but it would also be good to cross-link the validation tests the model passes with the respective figure/section where this is shown.

We thank you for this suggestion and agree it is a good idea, but it is indeed hard to fit these references to the figures without shortening the description of the properties we seek to validate models on to the point that some of them are impossible to understand.

l. 536: "On the otger hand, [...]."

Thank you for noticing this typo, we have now corrected it.

l. 582: What is meant by "inter-areal connectivity in V1"?

We have indeed made a mistake here and meant 'intra-areal connectivity in V1'.

l. 631: "The model makes five main simplifications: [...]"
The subsampling of synapses is left out here.

It was added to the list (see also earlier response to your comments).

Materials and Methods

l. 764: A spatial frequency should presumably be measured in inverse distance (or cycles per degree visual angle), not in Hz.

We corrected this mistake, thank you for pointing it out

l. 778: "Altogether it is reasonable to extrapolate from these number that as many as 50% of the synapses in layers 4 and 2/3 originate outside of V1."
This seems implausible given that Markov et al. (2011) showed in a retrograde tracing study that after injection into macaque V1 around 80% of the source neurons in the hemisphere are inside that area. Even though neurons may establish different numbers of synapses, 50% from outside the area seems altogether too high. What is the exact basis of this assumption?

As we describe in the Methods line 838-839 we are not aware of definitive data that quantify the proportion of extra-areal connections for cat V1 neurons (especially not in a layer resolved manner). The best data on this issue specifically for cat V1 we are aware of are from Stepanyants et al. 2009 who found that even for a cortical section of 800 um in diameter 76% of synapses come from outside of this region. If taken together with the fact that the bouton density rapidly falls off with distance from the neuron's soma (Buzás et al 2006), we think it is reasonable to extrapolate that as much as 50% percent of synapse come from outside V1.

That said, the Markov et al. (2011) is indeed compelling evidence that we potentially under-estimate the proportion of local connectivity. But it has to be acknowledged that this is data for macaques, not cat, and most importantly, unlike the Stepanyants study, Markov et al. rely on retrograde labeling where only the number of **pre-synaptic neurons**, and **not** directly the **synapses**, are measured. The issue with this is, that if local (portions of) axons contribute relatively fewer boutons than long-range (extra areal) axons, this would under-estimate the contribution of extra-aerial connectivity (because many synapses formed by one extra-areal pre-synaptic neuron would be counted as only one labeled extra-areal neuron). There is in fact a common pattern in pyramidal neurons which send their axons to other brain areas, whereby their axon, as it passes through the local substrate, provides few branches and synapses, but the main branching and bulk of synapses are only formed in its long-range target. We therefore advance that these species and methodological differences could explain the differences in the interpretation of the data between the Stepanyants et al. (2009) and Markov et al (2011) studies, and a definitive data on the exact proportion of local vs. extra-areal synapses are not yet available.

Nevertheless, we acknowledge that there is a possibility we under-estimate the proportion of local connections and we have therefore added a statement to the discussion acknowledging it and also added discussion of the potential impacts of this on our model (see lines 655-669 and more detailed discussion of this text in responses to your previous comments).

l. 787: "[...] rates of synaptic transmission (50%) [...]"
This seems to be taken from in vitro studies, but as the authors state themselves, the situation might be different in the presence of neuromodulators in vivo.

Indeed, but we are simply not aware of better data on this issue.

As we have already explained in greater detail in our previous responses, we now clearly acknowledge that we are potentially under-estimating the true number of local connections, and discuss the consequences in the discussion.

l. 857: "The functionally specific connectivity in V1 poorly understood."
This sentence lacks an "is".

We have rectified the error.

l. 882: "In Layer 2/3 excitatory neurons send long-range [...]"
The functional connectivity likelihood between pairs of neurons in Layer 2/3 depends only on the difference in preferred orientation. Does this connectivity scheme have a connection probability that decreases with distance or is confined to a certain patch of cortical surface?

The recurrent excitatory connectivity likelihood in Layer 2/3 has both a distance-dependent component (Described in subsection 6.1.4) and a functional component (Described in subsection 6.1.5), the latter depending indeed only on the difference in preferred orientation and not on distance.
We agree that this should have been stated more clearly in the introduction in the overview of the model, and we have therefore added the following sentence in lines 46-47

“The recurrent excitatory connectivity in Layer 2/3 has both a distance-dependent bias and a functional bias based on the orientation selectivity of the neurons”

Concerning the Material and Methods section. we believe that the following paragraph in subsection 6.1.3 already describes well how both components are combined together:

"The geometry of the cortico-cortical connectivity is determined based on two main principles: (i) the connection probability falls off with increasing cortical distance between neurons (Stepanyants et al. 2008, Buzás et al. 2006, Budd et al. 2001) (see Fig 1AB), and (ii) connections have a functionally specific bias (Buzás et al. 2006, Ko et al. 2011). The two principles are each expressed as a connection-probability density function, then multiplied and re-normalized to obtain the final connection probability profiles, from which the actual cortico-cortical synapses are drawn. The following two sections describe how the two probability density profiles of connectivity are obtained."

Moreover, we further explain in lines 922-923 and lines 955-957 that the long-range component of the distance-dependent excitatory connectivity in Layer 2/3 is paired with this functional bias.

In addition to this, we have now added a supplementary Table S1C which includes a summary of the different connectivity schemes of the model.

l. 885: The unit of sigma is missing.

Thank you for pointing that out, the unit is radians and we rectified this omission.

l. 907: "The weights of synapses formed by connections from Layer 4 to Layer 2/3 were set to 1, while the weights of synapses formed by the feedback connection from Layer 4 to Layer 2/3 were set to the same value as for the excitatory intra-laminar synapses."
The sentence is broken: it refers to "Layer 4 to Layer 2/3" two times.
Also the synaptic weight lacks a unit.
Further, why is one type of intra-laminar connection so strong, 1 (nS, presumably), and the other so much weaker, ~ 0.2 nS?

We apologize for this mistake and for the omission of the unit.

Concerning your remark on the strength difference of connections, we first want to emphasize that we are talking here about the feedforward connection from Layer 4 to Layer 2/3 which is not intra-laminar but inter-laminar. Nevertheless, this value might be indeed too high (relatively to the weights intra-laminar synapses), as we didn't find any data in the literature that could have helped us constraining it more. Partly due to the lack of functionally relevant external input from higher visual area, which targets preferentially the supragranular layers, the Layer 2/3 of our model needs this relatively higher drive from feedforward synapses to reach reasonable levels of spontaneous activity and make them responsive. Alternative mechanisms that could achieve this would be greater excitability of L2/3 vs L4 cells, but we didn't find any data on this either. The tradeoffs between modeling or not modeling the external drive were discussed in a previous response to your comments and included in the paper discussion.

To acknowledge this issue we have rewritten this sentence in the following way:

"The weights of synapses formed by connections from Layer 4 to Layer 2/3 were set to 1 nS. The data concerning the strength of feedforward inter-laminar synapses relative to intra-laminar synapses is lacking in V1, and we therefore acknowledge that this value might be too high. Nevertheless, such strong feedforward synapses to Layer 2/3 were necessary to drive these neurons sufficiently to compensate for the absence of feedback connections from higher visual areas. The weights of synapses formed by the feedback connection from Layer 2/3 to Layer 4 were set to the same value as for the excitatory intra-laminar Layer 4 synapses."

l. 921: "Basket cells. 2'3 form [...]"
Broken notation.
Thank you, we have fixed that.
l. 943: "We observed that the addition of this neuron-type-dependent delay factor improved the stability of the modeled cortical neural networks, reducing synchronous events during spontaneous activity."

This is not a new observation, see for example Brunel 2000.

We thank you for notifying us that other studies observed the same phenomenon. We did not claim here that we have discovered something new, we are just stating our observation, and we would be happy to reference a study that would support it. Nevertheless, after reading Brunel 2000, we understand that in this case it was not the introduction of different constant transmission delays for the different type of connections that enhanced the stability of the asynchronous state, but rather the sampling of each transmission delays from wide distributions, as shown by the following sentences in their subsection “5.2. Wide Distribution of Delays”:

“The analysis of the previous section can be generalized to the case in which delays are no longer fixed but are rather drawn randomly and independently at each synaptic site from a distribution Pr(D)

[...]

Thus a wide distribution of delays has a rather drastic effect on the phase diagram, by greatly expanding the region of stability of the asynchronous stationary state. The other regions that survive are qualitatively unmodified.”

Later in the article, the author describes a second model (Model B) for which excitatory and inhibitory connections have different characteristics, and notably different transmission delays distributions across E-E, E-I, I-E, and I-I connections, but he doesn’t report that this specific change has increased the stability of the asynchronous state of the network.

We therefore chose not to reference this study in our manuscript.

l. 972: Please give the parameter values.

We included the values for these parameters in Table S4 in the supplementary material that we added.

l. 1018: Was this the precomputed orientation preference or that measured in the model?

We apologize for not having written that clearly. We now specify that this was the precomputed orientation preference.

Eq. 6: It seems phi-phi_pref should be squared.

Thank you for pointing that out, we have now corrected it.

l. 1090: What were the specifics of the interpolation?
We now specify that this is a linear interpolation.

Reviewer #2:

We thank reviewer 2 for his warm support, and respond below in detail to his main query.

The paper reports on an impressive effort to combine many of the properties of the cat visual cortex, for which data is spread over several disparate sources, into a single, comprehensive model, and it argues convincingly for the importance of doing such detailed modeling work. The presentation of the model is well written, and readers will appreciate how clearly the model predictions are laid out, and how the experimental evidence, model assumptions and simplifications that went into constructing it are presented. I believe the paper will be valuable to anyone involved in simulating the visual cortex and for anyone interested in a mechanistic understanding of the various properties incorporated into the model. There is only one point in the paper that raised some concerns for me, detailed below.

The width of excitatory and inhibitory orientation tuning curves. In lines 154-155 and 413-414, the paper states that orientation tuning is only slightly broader for inhibitory neurons than it is for excitatory neurons. Focusing on L4 only, in the model the tuning HWHH is 21.1 vs 23.3 for excitatory and inhibitory neurons, so that inhibitory tuning curves are ~10% wider. But from what I could tell the sources indicate it should be much wider. The sources given were [8], [46], [47], and [65]. In source [47], Cardin et al 2007, they show in fig 4C and the corresponding text that in L4 the inhibitory tuning curves are ~5/3 times wider than excitatory ones on average. In sources [8] and [65], Finn et al 2007 and Nowak et al 2003, I could not find information specifically about the relative orientation tuning widths. In [46], Nowak et al 2007, they report in table 1 that inhibitory HWHH is ~4/3 times excitatory. That data is not layer specific, and they go on to speculate that the less selective inhibitory neurons they found came from L4, in which case the difference should be even larger in L4. If it is the case that I am reading these sources correctly, can you address the disparity between the model and data? Is it practical to tune the model parameters to match this data? If not, is it possible to identify why?

The reviewer is right that in the two studies we reference as source for inhibitory neuron tuning in V1 [46] and [47] (we do not cite [8] and [65] in this context) inhibitory neurons are more widely tuned relatively to excitatory one, than in the model (lines 158-161). Let us first start by being more explicit about the numbers. In our study we find that our layer 4 excitatory and inhibitory neurons have orientation tuning of 21.1 vs. 23.3 which corresponds to an 11 % increase for inhibitory neurons. In Cardin et al. 2007 they find that in layer 4 neurons the orientation tuning of regular spiking vs. fast spiking neurons is 14.5 vs 24.8 degrees, which is a 71% increase. In Nowak et al. indeed there is no layer specific data, but when pooled across all layers they find tuning width of exc vs. inh neurons as 23.4 vs. 31.9, which would correspond to 36% widening of tuning in inhibitory cells. On such a relatively basis these two studies seem to find a greater increase in the cat than in the model.

We would however like to point out that on the absolute basis, the increase is only about 10.3 degrees in Cardin et al. and 7.5 degrees in Nowak et al. We would argue that this is a relatively small amount given that the tuning can be anywhere between 0 and 90 degrees (and at least Nowak does find inhibitory untuned neurons (i.e. HWHH of 90)). Furthermore, consider that

Nowak reports SEM of ±9.2 and ±11.3 for the E and I neurons respectively, which are greater than the tuning width differences found, although Cardin et al. does report much smaller SEMs of ±1 and ±1.8 degrees (the difference in SEMs between the two studies raises in itself a question). Finally, note the differences of the absolute estimates of excitatory and inhibitory tuning width pooled across all layers between the two studies (Cardin et al. E:20.9 I:23 based on the supplementary table 1 vs. the Nowak et al E:23.4 I:31.9). These differences between studies, especially for inhibitory neurons, are of comparable magnitude to the absolute difference between E and I neurons found. The point we are trying to make is that given that level of uncertainty in the data both within studies and between the studies, and the small **absolute** differences between the E and I tuning width, it might be problematic to take the **relative** differences at face value, because just a small **absolute** deviations of the estimates from the true mean that might occur in the studies would lead to large deviations in the estimates of **relative** differences between E and I neurons.

That said, we think these two studies represent sufficient evidence for two 'qualitative' assertions: the tuning width of both excitatory and inhibitory neurons is quite narrow, (about 20 +/- 10 degrees) and the inhibitory neurons appear slightly more broadly tuned in absolute terms. We believe our model reproduces both these findings. We have reviewed the language with which we report these results (line 158-161) and we believe it is consistent with the existing evidence. We also have published experimental reports showing that the tuning width of excitatory and inhibitory conductances to intracellularly recorded V1 neurons are qualitatively similar (Figure 5 in Monier et al, 2003), offering further support for these qualitative claims.

In summary, our answer to the question 'Is it practical to tune the model parameters to match this data?' is a no. In the end to which of the results would we tune the model to - the 36% or 70%, (and as we explain, the true number can easily be substantially smaller or larger than both of those estimates).

All this said, we found the question of inhibitory neuron tuning interesting and ran a few experiments. As a courtesy to the reviewer we want to briefly share our findings.

We would like to start with a note, that the exact tuning width of the orientation tuning of inhibitory neurons in the model is a highly emergent property and no single model parameter directly controls it.

Sedigh-Sarvestani et al. 2017 found that the thalamo-cortical connections projecting to inhibitory neurons of cat V1 Layer differed from the ones projecting to excitatory neurons, such that receptive fields of the ON and OFF LGN neurons had a poor spatial overlap with the ON and OFF subregions of the receptive fields of the inhibitory neurons they project to, while such alignment was much better in excitatory neurons. In our model we ignore this difference, and we thought it could be the reason why layer 4 inhibitory neurons have HWHH that is only 11% higher than the excitatory neurons. We therefore modeled the reduced ON and OFF overlap in inhibitory neurons. This was partially successful in increasing the HWHH of the L4 inhibitory neurons, but only up to making it about ~20% broader than the HWHH of excitatory neurons. Additionally, this change also interferes with other properties we aim at reproducing such as size tuning and the unimodal distribution of the modulation ratio of the membrane potential.

Next we explored the hypothesis that increasing the weights of the E->I connections in Layer 4 would increase the HWHH of inhibitory neurons. This was in fact possible, and we could make inhibitory neurons to be 30 to 40% broader than the HWHH of excitatory neurons. Nevertheless, we had to use synaptic weights that were 10 times stronger which makes them highly unrealistic, and this change caused a large drop in the activity of the model, impacting its other properties.

As you can see the exact tuning width of inhibitory neurons in the model emerges from the model properties in a very complex way, and is tightly interwoven with a range of its other emerging properties. A proper in depth characterisation of this question would require substantial new amounts of work, beyond these initial exploratory experiments, that we feel are outside of the scope of this work. It would also further substantially enlarge already a very long manuscript. Yet, as we argued earlier, given the uncertainty in the existing data, it isn't even clear our model is in a significant quantitative disagreement with data (and the model qualitatively agrees with data). For all these reasons we propose not to include these preliminary findings into the manuscript.

Very minor comments/suggestions below.

1. Small typos on lines 445, 536

Thank you for pointing them out, we have now corrected them.

2. Table 1. I believe readers will find table 1 and the corresponding text to be a useful resource for getting a birds-eye view of comparable efforts made to model the visual cortex and to see all of the properties integrated into the model of this paper. Since some of the models are based on primate V1, it may be useful to mark out in table 1 for which ones this is the case. The reason for this suggestion is that it is not clear to me that all of the properties listed in table 1 apply equally to cat and primate models. Additionally, should row 3, labeled "Log-normally distributed...", contain open red circles in the same places as row 5? Lastly, I found the label for row 6 slightly confusing because several of the models model spontaneous activity. If there is a more specific label to use in that row, it could improve the table.

We thank you for this suggestion and inserted a dagger next to cat models and double daggers next to primate models.

We also added the following sentences in the caption of the table:

"When the species is clear, a dagger next to the model name means that the model was based on cat V1, whereas double daggers mark models based on primate V1."

We also modified the table so that row 3 also contain open red circles in the same places

The label in row 6 is specific to models that reproduce spontaneous states in which structured patterns of activity appear. We have modified it to make it more clear and it is now: “Structured patterns of spontaneous cortical activity”.

Reviewer #3:

We thank reviewer 3 for appreciating our work, and address his main concern below.

The paper of Antolik et al. presents a large-scale spiking network model for cat primary visual cortex that goes beyond previous network studies in many ways and provides interesting results. The paper encapsulates extensive work, seems competently done, and provides a new important contribution to the field. I am therefore positive to publication of the paper in the journal.

My only major comment relates to how this model can be used by the community. As I understand it the results presented in the paper all came from essentially the same set of (numerous) chosen model parameters. For the model to become a useful resource for the community, it is essential that users can test the model for other parameter choices.

While it is stated that the model will be available via v1model.arkheia.org, at the moment there is not much information here (only the figures shown in the paper, as I understand it). I assume that this will be updated in the process of publishing?
Further, the model is to be run via Mozaik, a tool that some of the authors have developed, but how this is done is not clear.
Maybe the authors could add a section illustrating the typical use of the model (for example, running it for new model parameters) for new users? Are there plans to make the model available via EBRAINS (where some of the authors are involved)?

One thing missing (as far as I can see) is the description of (i) on what computers the results were run and (ii) how long time it took to run the model on these computers.

Our manuscript indeed lacked instructions for running the model, we apologize for this. We have included the following sentence in the manuscript to resolve that:

"The model can be run by first installing Mozaik and its dependencies as indicated in the Mozaik Github repository, and then by downloading the implementation of model at https://github.com/CSNG-MFF/mozaik-models/ and following the instructions to run each experiment"

We believe that it is more appropriate for the instructions for how to run the model to be located in the README Github repository than in the manuscript itself. It also now contains an overview of each file it contains to help other users who want to use the model and modify some parameters. As you suggested, we have also added the specific description of the hardware we used to run the model as well as the typical run time for each experiment for our setup.

The Arkheia website for the model does not only contain the figures shown in the paper, but also the detailed lists of parameters of the model and of the experiments so that anyone can retrieve them as well as compare them with other V1 models. We have now modified it so that it also displays visually the stimuli that were shown to the model. We noticed that in the

introduction the following sentence potentially confused the reviewer in thinking that the Arkheia website contained also the code of the model:
"*To support collaborative knowledge integration, the model and all experimental protocols are implemented in an easily extensible simulation framework (Antolik et al. 2013), and all code and data are published online (http://v1model.arkheia.org).*"
We apologize for this, and we have now modified this sentence (line 35-40) so that it refers to the section of the manuscript that describes clearly that the code of the model is accessible via the following github repository: *https://github.com/CSNG-MFF/mozaik-models/*

The updated sentence now reads:

"To support collaborative knowledge integration, the model and all experimental protocols are implemented in an easily extensible simulation framework (Antolik et al. 2013). The complete model's code is published in an online repository (see Section 6), and all model parameters, stimuli and experimental protocols along with the analyzed results can also be explored online (see Section 5.1)."

Regarding EBRAINS:

For the moment we have registered the model in the EBRAINS Knowledge Graph: https://search.kg.ebrains.eu/instances/2791e32f-da43-468d-8cbe-9f8545647773.

We are working on integrating the Mozaik framework and the model into the EBRAINS software distribution, including registering the individual validation tests, however this is a more substantial project that is unlikely to be finished before publication of this manuscript.

Minor things:
- The recent paper by Billeh et al, Neuron (2020) on a comprehensive model for mouse visual cortex should be cited and the connection to the present work maybe discussed.

Thank you for reminding us about the more recent Allen Institute paper. This article is an iteration on the work from Arkhipov et al.2018, from the same research team, which we already discuss in our original manuscript (lines 566-576). In our revised version, we have therefore added the following two sentences to describe their most recent work (lines 568-579)

*"Finally, recent modeling studies from the Allen Institute (*Arkhipov et al. 2018, *Billeh et al. 2020)also followed a comprehensive, data-driven approach, employed a broad set of test protocols, and demonstrated orientation selectivity, amplification of thalamic inputs, presence of gamma oscillations, and log-normal distributions of firing rates. Unlike the present study they did not investigate more complex functional properties, such as size-tuning or trial-to-trial precision. Their models are complementary to the model presented here: cat V1 in the anesthetized state vs awake mice (*Arkhipov et al. 2018) *(respective merits of the two preparations are discussed in section 5.5). While Arkhipov et al. (*Arkhipov et al. 2018*) model was restricted only to Layer 4, Billeh et al. (Billeh et al. 2020) modeled all V1 layers. Both studies also incorporated neural morphology, although they showed that a simplified, integrate-and-fire version of their model*

remained in good agreement with in-vivo data, justifying the level of detail chosen in the present study."

- In Table 1, specific references should be listed when referring to Wielard series and the other series.

We thank you for this suggestion and have now added references for each of the series.

- On line 536: otger -> other
- On line 857: "is" is missing
- On line 921: I guess "2´3" is a typo?

We thank you for reporting these typos and we have now corrected them duly.

Other changes:

During the revision process we noticed a few other errors that we have now corrected:

Figure 6C included all neurons, even though discarded from the analysis due to too low activity or poor fitting results. In the revised version, this panel doesn't show the excluded neurons anymore.

Figure 6D showed only the positive RURA values. The histograms now display as well the neurons with negative RURA values. Note that these were already included when computing the population average values reported in the manuscript.

Line 1045: We noticed some errors in the mathematical formula of the Naka-Rushton functions. We have now corrected these formulas.

Line 1131: We originally wrote that 11% of the neurons were excluded from the HWHH analysis, as they weren't active enough or because the fitting with a gaussian was too poor. The number of excluded neurons is actually 7%, so we corrected this sentence.
