# Supplementary material for: A comprehensive data-driven model of cat primary visual cortex
Source: PLoS Comput Biol. 2024 Aug 21;20(8):e1012342. doi: 10.1371/journal.pcbi.1012342 (PMC11371232; doi:10.1371/journal.pcbi.1012342)
Supplement: Attachment — Submitted filename: Second response to reviewers.pdf [file pcbi.1012342.s010.pdf]

Our responses to reviewers' comments are in red. Changed text in the manuscript cited in our responses is in *italics*.

Reviewer #1:

We thank the first reviewer for his valuable input. Here are the replies to his remaining comments:

Could you please add labels to the vertical axis in Fig 2S?

Thank you for spotting this omission, we have now added the label for the vertical axis.

Sorry for being imprecise. What I meant is that I would have liked to see the fraction of cells with a SI greater than 0 or another threshold value. I think giving this value does not make your point weaker since to my understanding your results in that regard are compatible with the ones presented in Tailby et al.

We are sorry for the confusion, we have now included the percentage of cells with a suppression index greater than 0 in the following sentence (lines 277-279), comparing it with the values reported by Wang et al. (unfortunately Tailby et al. doesn't report similar analysis):

"94.1% of the cells of the model are suppressed for large stimuli as they display SI greater than 0, in great quantitative agreement with the value of 95% observed experimentally (Wang et al. 2009)."

Could you please clarify in the caption of Table 2 whether the rows are pre- or postsynaptic populations (and similarly for the columns)? One can get it from the context but it still would increase readability.

We agree that this would increase the clarity of the table. We have therefore added the following sentence at the end of the caption:
"Rows indicate pre-synaptic populations, whereas columns indicate post-synaptic populations."

Table S1:
You write under topology:
"Square 2D sheet using cortical distance" – To my understanding you use the Euclidean distance, I think "cortical distance" might be a bit confusing here.

The term 'distance' that we used originally was imprecise, as we wanted to express that whereas the LGN sheets are expressed in coordinates of degrees of visual space, the cortical sheets are expressed in millimeters of cortical space after application of the magnification factor. We have replaced the whole cell of the table by the following sentence, which should be more

clear:
“2D cartesian space using visual space coordinates (translated for V1 layers to cortical space coordinates through cortical magnification factor)”

Reviewer #2:

We are glad to read that the reviewer is satisfied with our answers, and we thank him for his comments during the previous round of reviews.

Reviewer #3:

We want to thank again the reviewer for his warm support.
