# Supplementary material for: A comprehensive data-driven model of cat primary visual cortex
Source: PLoS Comput Biol. 2024 Aug 21;20(8):e1012342. doi: 10.1371/journal.pcbi.1012342 (PMC11371232; doi:10.1371/journal.pcbi.1012342)
Supplement: Attachment — Submitted filename: Reviewer comments.pdf [file pcbi.1012342.s009.pdf]

Reviewer #2:

We thank reviewer 2 for his warm support, and respond below in detail to his main query.

The paper reports on an impressive effort to combine many of the properties of the cat visual cortex, for which data is spread over several disparate sources, into a single, comprehensive model, and it argues convincingly for the importance of doing such detailed modeling work. The presentation of the model is well written, and readers will appreciate how clearly the model predictions are laid out, and how the experimental evidence, model assumptions and simplifications that went into constructing it are presented. I believe the paper will be valuable to anyone involved in simulating the visual cortex and for anyone interested in a mechanistic understanding of the various properties incorporated into the model. There is only one point in the paper that raised some concerns for me, detailed below.

The width of excitatory and inhibitory orientation tuning curves. In lines 154-155 and 413-414, the paper states that orientation tuning is only slightly broader for inhibitory neurons than it is for excitatory neurons. Focusing on L4 only, in the model the tuning HWHH is 21.1 vs 23.3 for excitatory and inhibitory neurons, so that inhibitory tuning curves are ~10% wider. But from what I could tell the sources indicate it should be much wider. The sources given were [8], [46], [47], and [65]. In source [47], Cardin et al 2007, they show in fig 4C and the corresponding text that in L4 the inhibitory tuning curves are ~5/3 times wider than excitatory ones on average. In sources [8] and [65], Finn et al 2007 and Nowak et al 2003, I could not find information specifically about the relative orientation tuning widths. In [46], Nowak et al 2007, they report in table 1 that inhibitory HWHH is ~4/3 times excitatory. That data is not layer specific, and they go on to speculate that the less selective inhibitory neurons they found came from L4, in which case the difference should be even larger in L4. If it is the case that I am reading these sources correctly, can you address the disparity between the model and data? Is it practical to tune the model parameters to match this data? If not, is it possible to identify why?

The reviewer is right that in the two studies we reference as source for inhibitory neuron tuning in V1 [46] and [47] (we do not cite [8] and [65] in this context) inhibitory neurons are more widely tuned relatively to excitatory one, than in the model (lines 158-161). Let us first start by being more explicit about the numbers. In our study we find that our layer 4 excitatory and inhibitory neurons have orientation tuning of 21.1 vs. 23.3 which corresponds to an 11 % increase for inhibitory neurons. In Cardin et al. 2007 they find that in layer 4 neurons the orientation tuning of regular spiking vs. fast spiking neurons is 14.5 vs 24.8 degrees, which is a 71% increase. In Nowak et al. indeed there is no layer specific data, but when pooled across all layers they find tuning width of exc vs. inh neurons as 23.4 vs. 31.9, which would correspond to 36% widening of tuning in inhibitory cells. On such a relatively basis these two studies seem to find a greater increase in the cat than in the model.

We would however like to point out that on the absolute basis, the increase is only about 10.3 degrees in Cardin et al. and 7.5 degrees in Nowak et al. We would argue that this is a relatively small amount given that the tuning can be anywhere between 0 and 90 degrees (and at least Nowak does find inhibitory untuned neurons (i.e. HWHH of 90)). Furthermore, consider that

Nowak reports SEM of ±9.2 and ±11.3 for the E and I neurons respectively, which are greater than the tuning width differences found, although Cardin et al. does report much smaller SEMs of ±1 and ±1.8 degrees (the difference in SEMs between the two studies raises in itself a question). Finally, note the differences of the absolute estimates of excitatory and inhibitory tuning width pooled across all layers between the two studies (Cardin et al. E:20.9 I:23 based on the supplementary table 1 vs. the Nowak et al E:23.4 I:31.9). These differences between studies, especially for inhibitory neurons, are of comparable magnitude to the absolute difference between E and I neurons found. The point we are trying to make is that given that level of uncertainty in the data both within studies and between the studies, and the small absolute differences between the E and I tuning width, it might be problematic to take the relative differences at face value, because just a small absolute deviations of the estimates from the true mean that might occur in the studies would lead to large deviations in the estimates of relative differences between E and I neurons.

That said, we think these two studies represent sufficient evidence for two 'qualitative' assertions: the tuning width of both excitatory and inhibitory neurons is quite narrow, (about 20 +/- 10 degrees) and the inhibitory neurons appear slightly more broadly tuned in absolute terms. We believe our model reproduces both these findings. We have reviewed the language with which we report these results (line 158-161) and we believe it is consistent with the existing evidence. We also have published experimental reports showing that the tuning width of excitatory and inhibitory conductances to intracellularly recorded V1 neurons are qualitatively similar (Figure 5 in Monier et al, 2003), offering further support for these qualitative claims.

In summary, our answer to the question 'Is it practical to tune the model parameters to match this data?' is a no. In the end to which of the results would we tune the model to - the 36% or 70%, (and as we explain, the true number can easily be substantially smaller or larger than both of those estimates).

All this said, we found the question of inhibitory neuron tuning interesting and ran a few experiments. As a courtesy to the reviewer we want to briefly share our findings.

We would like to start with a note, that the exact tuning width of the orientation tuning of inhibitory neurons in the model is a highly emergent property and no single model parameter directly controls it.

Sedigh-Sarvestani et al. 2017 found that the thalamo-cortical connections projecting to inhibitory neurons of cat V1 Layer differed from the ones projecting to excitatory neurons, such that receptive fields of the ON and OFF LGN neurons had a poor spatial overlap with the ON and OFF subregions of the receptive fields of the inhibitory neurons they project to, while such alignment was much better in excitatory neurons. In our model we ignore this difference, and we thought it could be the reason why layer 4 inhibitory neurons have HWHH that is only 11% higher than the excitatory neurons. We therefore modeled the reduced ON and OFF overlap in inhibitory neurons. This was partially successful in increasing the HWHH of the L4 inhibitory neurons, but only up to making it about ~20% broader than the HWHH of excitatory neurons.

Additionally, this change also interferes with other properties we aim at reproducing such as size tuning and the unimodal distribution of the modulation ratio of the membrane potential.

Next we explored the hypothesis that increasing the weights of the E->I connections in Layer 4 would increase the HWHH of inhibitory neurons. This was in fact possible, and we could make inhibitory neurons to be 30 to 40% broader than the HWHH of excitatory neurons. Nevertheless, we had to use synaptic weights that were 10 times stronger which makes them highly unrealistic, and this change caused a large drop in the activity of the model, impacting its other properties.

As you can see the exact tuning width of inhibitory neurons in the model emerges from the model properties in a very complex way, and is tightly interwoven with a range of its other emerging properties. A proper in depth characterisation of this question would require substantial new amounts of work, beyond these initial exploratory experiments, that we feel are outside of the scope of this work. It would also further substantially enlarge already a very long manuscript. Yet, as we argued earlier, given the uncertainty in the existing data, it isn't even clear our model is in a significant quantitative disagreement with data (and the model qualitatively agrees with data). For all these reasons we propose not to include these preliminary findings into the manuscript.

Thank you for your very comprehensive reply. I am satisfied that the tuning curves in the model sufficiently match the data for the purposes of the present work.

Very minor comments/suggestions below.

1. Small typos on lines 445, 536

Thank you for pointing them out, we have now corrected them.

2. Table 1. I believe readers will find table 1 and the corresponding text to be a useful resource for getting a birds-eye view of comparable efforts made to model the visual cortex and to see all of the properties integrated into the model of this paper. Since some of the models are based on primate V1, it may be useful to mark out in table 1 for which ones this is the case. The reason for this suggestion is that it is not clear to me that all of the properties listed in table 1 apply equally to cat and primate models. Additionally, should row 3, labeled "Log-normally distributed...", contain open red circles in the same places as row 5? Lastly, I found the label for row 6 slightly confusing because several of the models model spontaneous activity. If there is a more specific label to use in that row, it could improve the table.

We thank you for this suggestion and inserted a dagger next to cat models and double daggers next to primate models.

We also added the following sentences in the caption of the table:

"When the species is clear, a dagger next to the model name means that the model was based on cat V1, whereas double daggers mark models based on primate V1."

We also modified the table so that row 3 also contain open red circles in the same places

The label in row 6 is specific to models that reproduce spontaneous states in which structured patterns of activity appear. We have modified it to make it more clear and it is now: “Structured patterns of spontaneous cortical activity”.

Thank you for the changes, and for your attention to my concerns.
